# Nuances of Gender Identity for a Transgender Patient Receiving Inpatient Treatment for Paranoid Schizophrenia: A Case Study

**DOI:** 10.1155/crps/5529934

**Published:** 2026-01-11

**Authors:** Ravleen Kaur Suri, Kathleen P. Heslin, Susan Sperry, Luba Leontieva

**Affiliations:** ^1^ Department of Psychiatry and Behavioral Sciences, State University of New York Upstate Medical University, Syracuse, 13210, New York, USA, upstate.edu

**Keywords:** case report, gender dysphoria, psychosis, schizophrenia, transgender

## Abstract

**Introduction:**

Nearly one‐fourth of individuals diagnosed with schizophrenia may experience symptoms of gender dysphoria. Patients may identify with a gender other than the gender assigned at birth before or after psychotic disorder onset. In either case, the presence of both psychosis and gender dysphoria may complicate evaluation and treatment.

**Case:**

We present the case of a 45‐year‐old patient with a past psychiatric history of paranoid schizophrenia who was assigned male at birth and began to identify as a woman in her youth around the age when her psychosis first developed. During the admission discussed in this report, the patient identified as a man during a period with frank psychotic symptoms but preferred feminine clothing and structured the clothes to create the appearance of female anatomy. Later in admission, as psychosis resolved with pharmacologic treatment, the patient began to identify as a woman and adopted a traditionally feminine name and female‐coded dress. Psychiatric assessment, record‐gathering, and psychological assessment were completed to better understand her perspective on self and others and the historical context of her symptoms and gender identity.

**Conclusion:**

This case highlights the risk of assessing gender identity in patients with active thought disorders. Patients’ perspectives on gender and presentation may shift during periods of psychosis compared to periods of remission. Assessment and treatment of gender dysphoria is possible and beneficial in patients with psychotic disorders, but physicians must be cautious about diagnosing and treating gender dysphoria during active psychosis.

## 1. Introduction

Despite growing awareness of unique mental health concerns in transgender and gender‐nonconforming patients, research on psychotic disorders in this population remains limited, and understanding of best practices for treating these patients in inpatient settings is developing.

The Diagnostic and Statistical Manual of Mental Disorders, fifth edition, text revision (DSM‐5‐TR) defines gender dysphoria as “a marked incongruence between one’s experienced/expressed gender and assigned gender” [1], while the International Classification of Diseases, 11th revision (ICD‐11) recognizes *gender incongruence of adolescence and adulthood* as “a marked and persistent incongruence between an individual’s experienced gender and the assigned sex, which often leads to a desire to “transition,” in order to live and be accepted as a person of the experienced gender, through hormonal treatment, surgery, or other health care services to make the individual’s body align, as much as desired and to the extent possible, with the experienced gender” [2]. Discussion continues regarding the risks of using terms such as *gender dysphoria* and *incongruence*, including over‐pathologizing and medicalization of identity fluidity, and uncertainty over the classification of gender dysphoria as a true mental disorder [3]. However, assignment of *gender dysphoria* and *gender incongruence* is often required for patients to access related healthcare [4]. For the purpose of this report, although imperfect, *gender dysphoria* will be used to represent the experience of discrepancy between identity and sex assigned at birth.

In the general population, the prevalence of gender dysphoria is one per 100,000 women and one per 30,000 men; the prevalence of schizophrenia is 1%, which suggests the population of people who experience both is small [5]. Up to 25% of people with schizophrenia may have delusional views about gender and physical characteristics [6]. Patients with preexisting gender dysphoria sometimes only describe their symptoms after psychotic symptoms appear [7], which may complicate diagnosis and treatment. Recent studies suggest between 0.39% and 2.7% of the US population identifies as transgender or nonbinary [8]. Diagnoses of schizophrenia distribute bimodally; most cases present around age 22 years and some around the age of 46 years [9]. Some research suggests that gender dysphoria first arises between ages 3 and 7 years, and individuals experience persistent gender dysphoria into adulthood before initiating transition care [10]. As psychosis may emerge in young adulthood when issues of identity and gender dysphoria are paramount, further understanding of how to diagnose and conceptualize psychosis and better understand gender identity issues in this population is needed.

Some research suggests a higher rate of psychosis in transgender patients on inpatient units [11] and a higher prevalence of psychotic disorders in this population [12], while other work suggests transgender people have rates of schizophrenia equal to the general population [13]. Variability may be related to differences in study design, including insufficient accounting for demographic factors, inconsistent recruitment techniques, and varied strategies for defining transgender identity [14]. Studies may account for only part of the transgender population by relying on a formal ICD‐10 diagnosis of gender dysphoria rather than self‐reported gender identity, or by including transgender patients but excluding gender‐nonconforming or nonbinary individuals [14].

There are limited published case studies on schizophrenia in transgender patients. One study [15] describes a male patient who began dressing in female clothes at age seven and worked on an all‐female farm. In his 30s, he developed schizophrenia, experienced psychiatric hospitalizations, and remained committed to female dress. Another describes a male patient with schizoaffective disorder, mixed type, who endorsed intent to transition after smoking cannabis and reading about transgenderism [16]; after psychiatric stabilization, his ideas about transitioning dissipated. In a recent case, a patient diagnosed with schizoaffective disorder, bipolar type, identified as female only during psychotic episodes [17]. Other studies have described religiously focused psychosis in a transgender and intersex individual [18] and cases of gender dysphoria that presented before the onset of psychotic symptoms but were reported only after patients began treatment for psychosis [7].

We present the case of a 45‐year‐old African American patient with a history of paranoid schizophrenia and a decades‐long history of dressing as female who presented with auditory hallucinations, then disclosed identification with the opposite gender after treatment for psychosis and improvement of psychiatric status.

## 2. Case Presentation

The patient was a 45‐year‐old African American unhoused biological male with a past psychiatric diagnosis of paranoid schizophrenia and prior inpatient hospitalizations, with no known suicide attempts, who presented to the hospital’s emergency department (ED) with reported auditory hallucinations. In the six months preceding admission, this patient had presented to the ED and the local comprehensive psychiatric emergency program 37 times, lost his medications, and failed to follow up outpatient. A month before, he had been admitted to a separate acute psychiatric unit for command hallucinations ordering injury to self. Three days earlier, he had been evaluated in the ED for paranoid thoughts of being pursued by a serial killer and was discharged to outpatient care. At the presentation discussed in this report, he denied paranoid thinking, reported new‐onset hallucinations, and displayed constricted affect. During the interview, he was dressed in casual gender‐nonspecific clothing and wore a curly long‐haired wig. He was voluntarily admitted to the inpatient unit for psychotic symptoms.

Initially, he was cooperative but guarded, internally preoccupied, and disorganized, with minimal eye contact and mumbling speech. When asked early in treatment, he stated he was male and denied transgender identity. However, from the first day of admission, he wore a bra stuffed with material to create the contour of breasts; the size of the bra changed throughout the day. After treatment initiation, he reported cessation of hallucinations but remained internally preoccupied and isolated with an impoverished thought process.

Toward the end of this admission, after significant psychiatric stabilization, the patient expressed identification with the female gender (she/her) and a preference for a traditionally female name (the patient will be addressed using she/her pronouns from this point forward). She reported being most comfortable when dressed as a female and stated attraction to both women and men. At the time of discharge, the patient identified with the female sex.

### 2.1. Family History

Per the patient report, there was no significant medical or psychiatric familial history.

### 2.2. Early Life and Personal History

Per the patient’s report, she was the youngest of two brothers and one sister; her parents separated early in her childhood, and she did not recall her father. Early in life, she had no desire to wear women’s clothing and no attraction to men. She reported no childhood trauma, abuse, or bullying but cited poor memory of her childhood. She shared that growing up, she was bright, good at “spelling bee competitions,” and liked mathematics. She had a close circle of friends and obtained a General Educational Development (GED) high school equivalency credential.

Records from the patient’s previous forensic and inpatient psychiatric hospitalizations indicated a significant arrest history starting at 16 years old, including second‐degree rape, weapon possession, harassment, and unauthorized use of a vehicle. She was hospitalized at a state facility several times for persecutory delusions (specifically, fear of others poisoning food and medications), bizarre behavior, threats to others, and intermittent suicidal ideation. During an early admission, she was observed wearing women’s clothing and shoes, and she stated that she dressed as a female to protect herself from others.

She worked for a moving and packing company, then was incarcerated for 4 years after having sexual relations with an underage female prostitute at age 20 years. In jail, she resided in the male unit. At the age of 21 years, she began hearing multiple male “ghostly” voices and experienced paranoid ideation, including a belief that serial killers in jail wanted to hurt her. She discussed her paranoia with a counselor in jail, after which she was referred to a forensic psychiatric facility. Around this time, she first experienced the desire to dress as a woman and the perception that she looked “funny” in men’s clothing. Due to limited clothing options, she was forced to suppress the urge to dress in women’s clothing.

After being released from jail, she lived with her mother and sister. Due to symptom recurrence, she was admitted to a long‐term psychiatric facility several times in the following decades. Between hospitalizations, she began to wear her mother’s and sister’s high heels, skirts, underwear, and brassieres. The patient associated this partly with “boredom” but also endorsed a sense of happiness when dressing as a woman. She began braiding her hair and openly wearing tight feminine clothing. She recognized no association between the shift in gender identity and her psychotic symptoms.

When asked about her initial reticence around her gender identity during the admission discussed in this report, she stated that providers had not asked “the right questions.” She denied distress or impairment related to the incongruence between her assigned gender and preferred female identity; instead, she endorsed increased satisfaction with sense of self when presenting as female. She disclosed having sexual relations with both men and women in the past. She reported exclusive interest in long‐term relationships with women, though she related this to a desire to avoid infidelity that she ascribed to male “boredom” in relationships. She endorsed interest in limited gender affirmation surgery, that is, breast and buttock augmentation, without genital reconstruction.

### 2.3. Previous Medication and Drug Use

During historical admissions, the patient’s psychotic symptoms had been treated with oral haloperidol 10 mg daily and olanzapine of unknown dose. Three months prior to admission, the patient had received intramuscular long‐acting injectable (LAI) first 100 and then 50 mg of haloperidol decanoate. During a separate admission a month earlier, the patient was treated with and discharged on risperidone 2 mg twice daily. Shortly after that discharge, the patient lost and discontinued this medication.

Per records from historical long‐term psychiatric admissions, the patient had a distant history of substance use. The patient denied recent substance use throughout this admission. However, a urine toxicology screen prior to the current admission was positive for fentanyl.

### 2.4. Pharmacologic Intervention

Initially, the patient was restarted on risperidone 2 mg twice daily. After 4 days, she denied auditory or visual hallucinations and paranoia but continued to demonstrate internal preoccupation and impoverished thinking. She expressed interest in restarting haloperidol LAI and transitioned to haloperidol 5 mg twice daily on the sixth day of admission. Haloperidol was continued until discharge. Haloperidol decanoate 100 mg intramuscular injection was administered 2 days before discharge. She was discharged on an overlap regimen of haloperidol 5 mg twice daily, with a plan for administration of 100 mg haloperidol decanoate every 28 days. During this admission, a previously present intermittent chin tremor and rare right upper extremity tremor were noted, though the patient denied any associated distress. The patient was started on benztropine 1 mg twice daily for extrapyramidal symptoms.

### 2.5. Psychological Findings

The patient underwent a brief psychological assessment by a psychologist in which she was administered seven cards from the Thematic Apperception Test (TAT) [19, 20] cards 13B, 7 GF, 17 BM, 9 GF, 3 BM, 18 BM, and 16 to further evaluate her diagnostically and to better understand her sense of self, others, and the world. The stories she told were organized and linear without bizarre or unusual content. Her stories tended to be vague, impoverished, and perseverative. She had some difficulty identifying characters’ emotions and instead reported what characters were thinking. Additional queries did not improve the response quality. When she did identify emotions, she described characters as boring, anxious, or sad. In one story, she described the main character as having strong emotions while pursuing a goal but could not clarify those emotions. She used pronouns consistently in her stories and quickly identified characters as female or male. In three stories, she described characters as longing for home or figuring out how to get home. Several stories focused on wondering where a parent was or waiting for a parent or parents to return. A parent featured in one story worried about her child and wondered what she was “up to.” In three stories, characters were thinking about what to do with their day and how to enjoy themselves. Characters appeared isolated, with no descriptions of interactions, collaboration, or support among them. Across all stories, problems were poorly defined, and there was no clear path to problem‐solving. Stories tended to end positively; children were reunified with parents, characters achieved goals, and characters found their way home. However, these happy endings occurred as a matter of course, rather than as the result of characters’ efforts. These impoverished narratives reflected the patient’s underlying primary psychotic disorder, while the themes suggested limited emotional awareness, social isolation, longing for connection, and desire for positive experiences.

## 3. Discussion

Select cases describe patients whose psychotic symptoms appeared after the onset of gender dysphoria. In the 2017 case series by Meijer et al. [7], all four patients experienced initial signs of gender dysphoria before the age of 12 years and developed psychosis later in life. The risk of psychosis in transgender individuals may be heightened by stressors like discrimination, marginalization, trauma, and social stigma [14]; these are social stressors which may lead to endocrine and inflammatory changes associated with vulnerability to psychosis [21]. Most other work describes patients whose gender identity shifts after psychosis onset. A 2022 report describes an assigned‐male‐at‐birth patient diagnosed with schizoaffective disorder who identified as a woman during psychotic breaks but returned to a male identity upon stabilization with pharmacologic interventions [17].

Our case describes an individual whose transgender identity developed around the same time as her psychotic symptoms. In periods of acute illness, her gender identity and presentation fluctuated. However, her sense of self and preference for dress aligned with female identity, and her interest in gender‐affirming care reemerged after pharmacologic treatment for psychosis and resolution of active symptoms. Her sense of self as female appears to have been present and relatively stable for most of her adult life.

Based on the limited case history, it is unclear whether this patient’s psychosis in youth influenced the formation of her gender identity. The patient’s main presenting psychotic symptoms resolved, but some internal preoccupation and thought impoverishment remained. It is unknown how this patient’s experience of gender identity presents when she lacks psychotic symptoms. Additional follow‐up would be essential to confirm that her female gender identity is stable during periods of minimal or no symptoms.

In cases like our patient’s, establishing a diagnosis of gender dysphoria is not the primary focus, but understanding the history of dysphoria or gender identity may help case conceptualization. If a patient only experiences apparent dysphoria when demonstrating active psychotic symptoms, then belief in a different gender identity may represent delusional thinking and indicate an opportunity for psychiatric intervention. Conversely, if a patient retains that gender identity when lacking psychotic symptoms, then it may be most appropriate to treat the patient as a trans or gender‐nonconforming individual who also has psychotic symptoms; in this scenario, a diagnosis of gender dysphoria could be warranted if the patient wishes to access related medical care or to continue gender‐affirming pharmacotherapy inpatient.

This case presents an opportunity to consider accommodations for transgender and gender‐nonconforming patients and those with gender dysphoria in the inpatient psychiatric setting. Previous literature, State of New York Office of Mental Health guidelines, and World Professional Association for Transgender Health guidelines suggest individualized adaptations including using gender‐affirming names and pronouns, exploring risk factors, including safety in the home environment, incorporating affirming lifestyle components like binders and shaving accessories if safe, providing rooming accommodations specific to gender identity rather than assigned sex, considering the risk of violence toward the patient from others, providing access to relevant medical care and hormone therapy without interruption, and considering on a case‐by‐case basis whether a patient would benefit more from having a single room and risking isolation or sharing a room and risking discomfort with lack of privacy [22–24]. Other suggestions include system changes such as staff training on bias toward LGBTQIA+ (lesbian, gay, bisexual, transgender, queer/questioning, intersex, asexual, plus related identities) individuals, consistent use of patients’ pronouns, affirming language on forms, gender‐neutral bathrooms, and avoidance of stigmatizing language in documentation [23, 25].

Furthermore, there is nuance to pharmacotherapy for patients receiving gender‐affirming therapy, including hormone replacement. Several current guidelines advise continuing hormone replacement therapy during admission [22, 24] and previous work recommends referring to Endocrine Society guidelines [26]. Puberty suppressants, i.e., gonadotropin‐releasing hormone agonists, pose a risk of QTc prolongation, metabolic effects, seizure risk, and lower bone mineral density, while estrogen risks weight gain and dyslipidemia, and testosterone is associated with aggression, liver toxicity, and other effects [27]. These side effects are significant for patients receiving psychotropic medications like antipsychotics or mood stabilizers, which carry similar risks. Estrogen and testosterone may interact with psychotropic medications like lamotrigine or fluvoxamine and bupropion, respectively, through cytochrome induction or inhibition. Careful review of medication lists is critical to minimize toxicity and avoid undertreatment.

## 4. Conclusions

To ensure equity in clinical care, awareness of and sensitivity to gender identity issues is paramount. As psychotic disorders may present with fluctuations in sense of self, including gender, clinicians must be cautious about diagnosing gender dysphoria in the context of psychosis. Decisions about initiating gender‐affirming care should not be made during periods of acute illness [17]. However, clinicians would benefit from acknowledging, validating, and supporting patients through periods when sense of self is in flux. Acceptance paired with, as our patient noted, the willingness to “ask the right questions” may build therapeutic alliance in a way that enhances patient care overtime.

## Consent

The authors obtained informed written consent from the patient involved in this case study. This consent form is available for review upon request. In this report, the pronouns used to refer to the patient shift according to the pronouns the patient used as treatment progressed.

## Disclosure

Ravleen Kaur Suri was affiliated with the Department of Psychiatry and Behavioral Sciences at State University of New York Upstate Medical University at the time of the work contained in this report. She is currently affiliated with the Department of Psychiatry at BronxCare Health System, Bronx, NY, 10457.

## Conflicts of Interest

The authors declare no conflicts of interest.

## Author Contributions

Ravleen Kaur Suri and Kathleen P. Heslin were responsible for project conceptualization, project administration, drafting, and final review and editing. Susan Sperry and Luba Leontieva were responsible for project conceptualization, final review and editing, and project supervision.

## Funding

The authors received no funding for this research.

## Data Availability

The case report data used to support the findings of this study are included within the article.
